# Supplementary material for: Dietary Amino Acid Composition and Glycemic Biomarkers in Japanese Adolescents
Source: Nutrients. 2024 Mar 19;16(6):882. doi: 10.3390/nu16060882 (PMC10975557; doi:10.3390/nu16060882)
Supplement: Supplementary file 1 [file nutrients-16-00882-s001.zip › Supplementary_FiguresS1-S4.pdf]

Supplementary Figures S1–S4

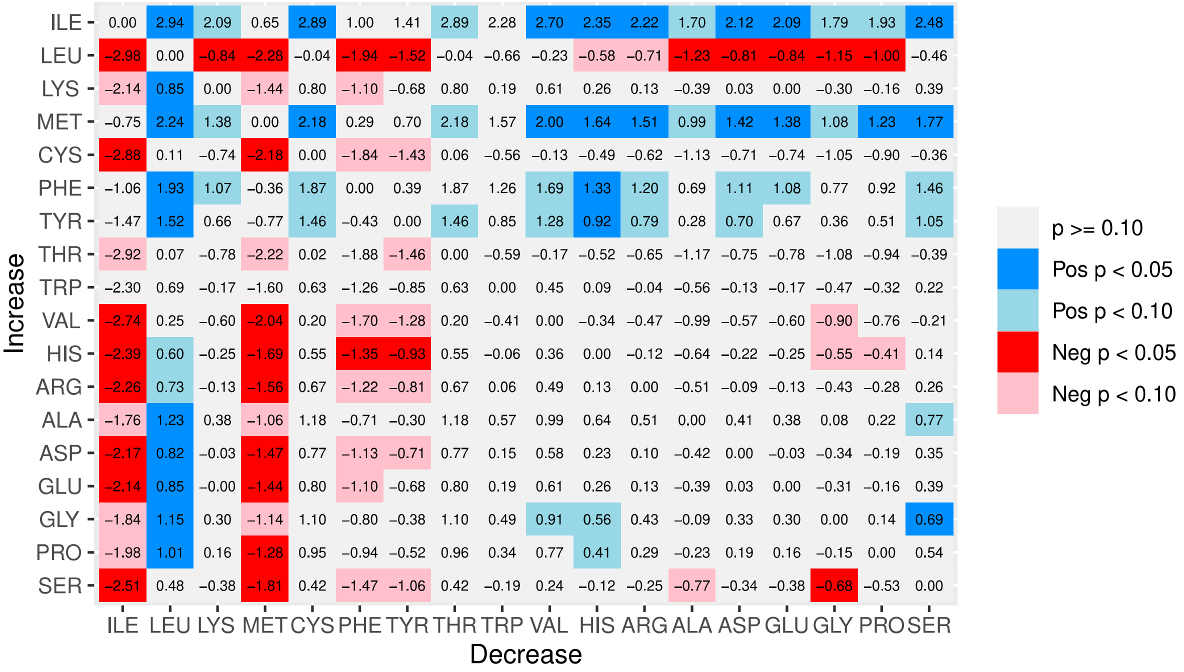

Figure S1. Effects of one-to one replacement of 0.1% of total amino acids on glucose (mg/dl). Amino acids in row (increase) were substituted for amino acids in columns (decrease). Pos, positive effect; Neg, negative effect.

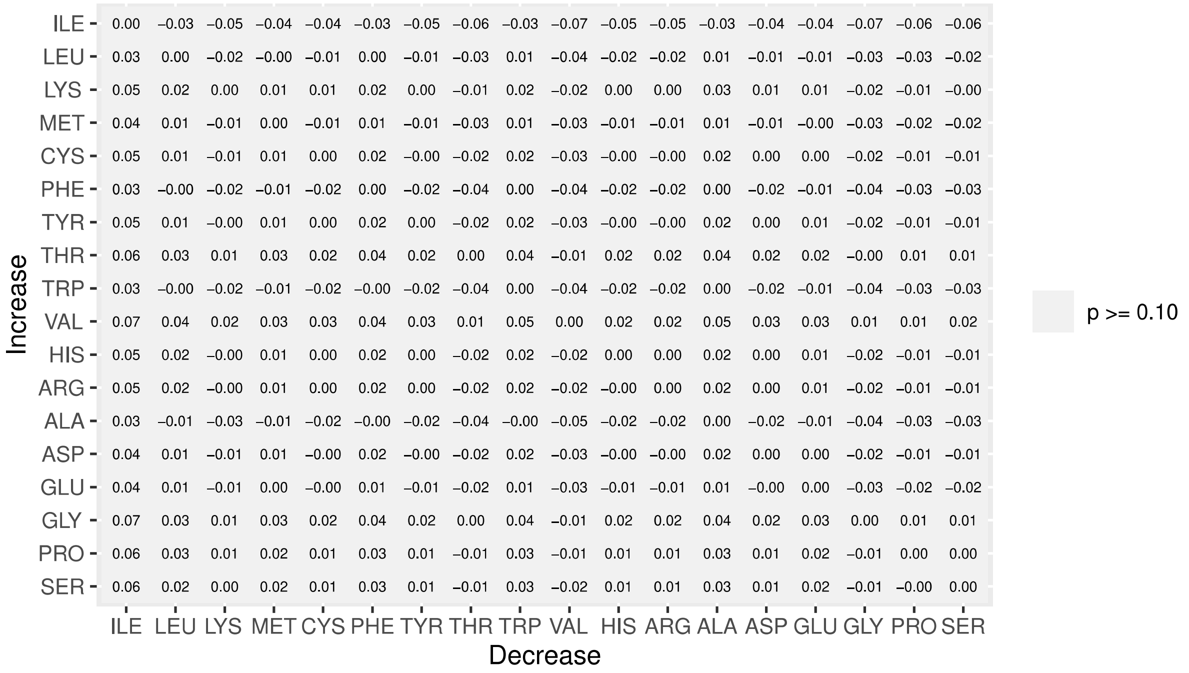

Figure S2. Effects of one-to one replacement of 0.1% of total amino acids on natural-log-transformed insulin (µU/ml). Amino acids in row (increase) were substituted for amino acids in columns (decrease).

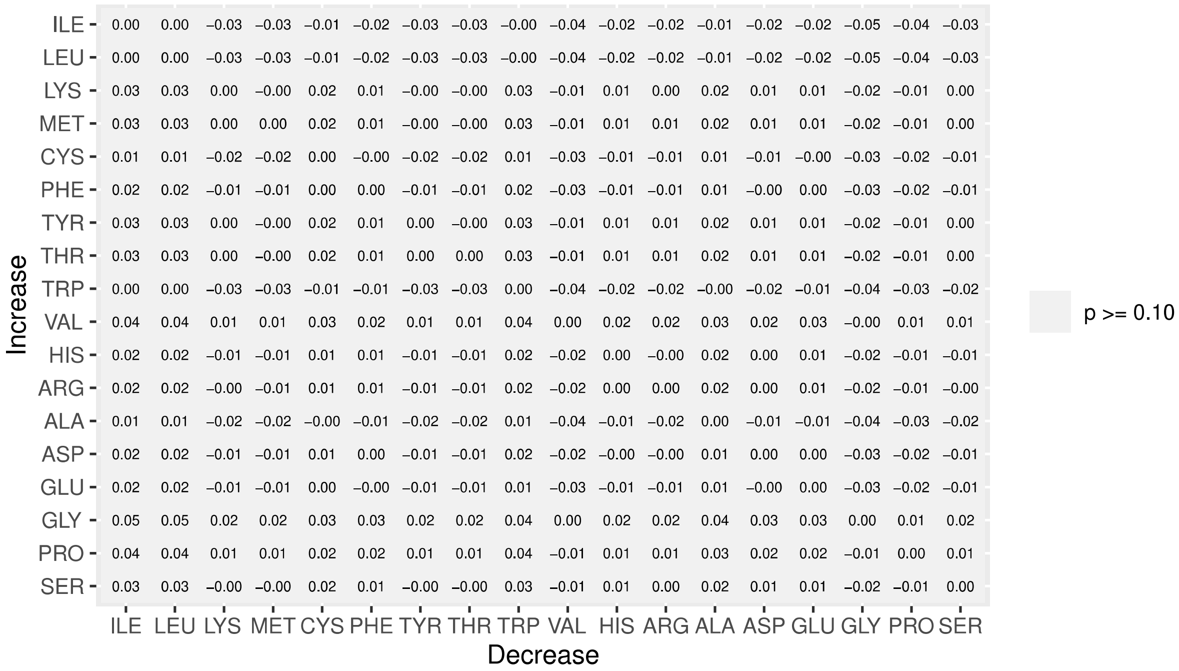

Figure S3. Effects of one-to one replacement of 0.1% of total amino acids on the natural-log-transformed homeostatic model assessment for insulin resistance. Amino acids in row (increase) were substituted for amino acids in columns (decrease).

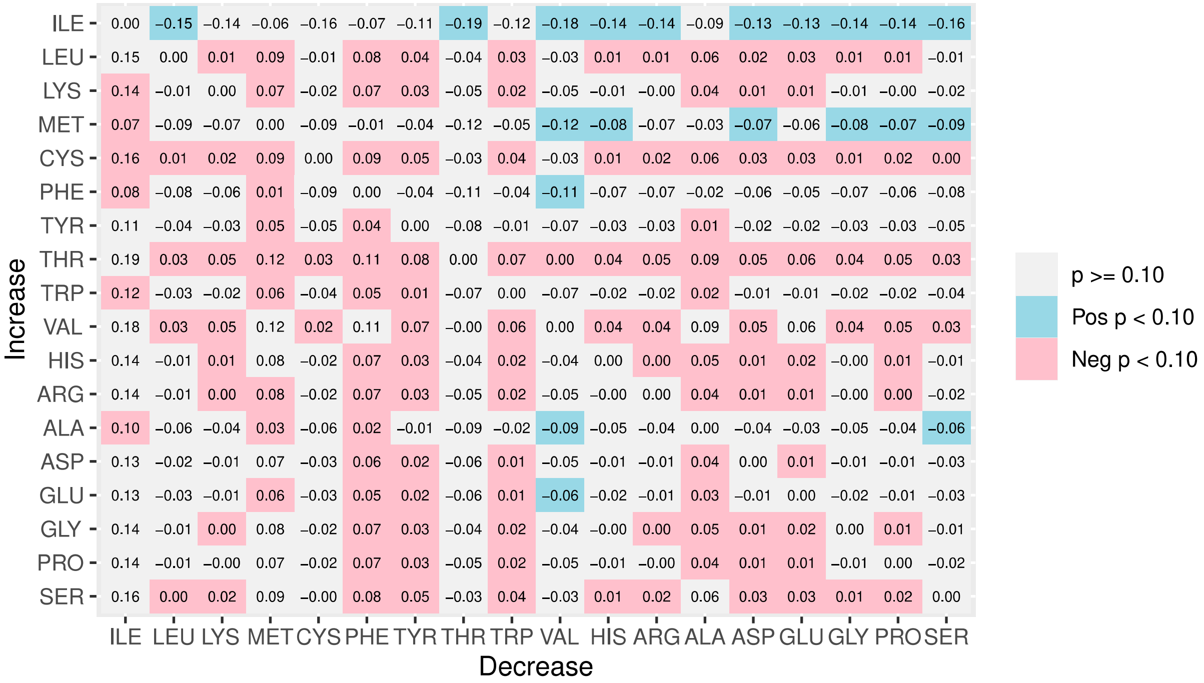

Figure S4. Effects of one-to one replacement of 0.1% of total amino acids on the natural-log-transformed homeostatic model assessment for ß cell function (%). Amino acids in row (increase) were substituted for amino acids in columns (decrease). Pos, positive effect; Neg, negative effect.
