# Supplementary material for: Dietary Amino Acid Composition and Glycemic Biomarkers in Japanese Adolescents
Source: Nutrients. 2024 Mar 19;16(6):882. doi: 10.3390/nu16060882 (PMC10975557; doi:10.3390/nu16060882)
Supplement: Supplementary file 1 [file nutrients-16-00882-s001.zip › Supplementary_TableS1.pdf]

Supplementary Table S1. R Script for calculate replacement.

```
library(tydyverse)
library(composisionts)

# list of amino acids
aminoacids <- c( "LEU",   "LYS",   "MET",   "CYS",
               "PHE",   "TYR",   "THR",   "TRP",   "VAL",
               "HIS",   "ARG",   "ALA",   "ASP",   "GLU",   "GLY",
               "PRO",   "SER",   "ILE"   )

# function to calculate the predicted effect of 0.1% replacement
replace.compo <- function(lmAA, compMean){
  # lmAA is the result of multivariate linear regression function
  # compMean is the closure of the means
  meandat <- slice_head(lmAA$model, n=1)[,-1]  # format the table for the means
  meandat$ilrAA[,1:17] <- ilr(compMean)        #  insert the means
  # "covariates" is the list of means of covariates
  meandat[,2:13] <- as.numeric(covariates)     #
  # predicted values for the means
  fit.mean <- predict(lmAA, newdata=meandat, se.fit=TRUE)
  # 0.1% replacement
  for(j in 1:18) {
    offsetMean <-                              # one-to-all replacement
      acomp(as.numeric(compMean) +             # the former is inactive
            c(0.001, rep(-(0.001/17), 17)))    #  this results the same effect
                                               #    in all columns
    offsetMean <-                              # one-to-one replacement
      acomp(as.numeric(compMean) +             # the latter is active
            c(0.001, rep(0, 17)) +             #
            c(rep(0, j - 1), -0.001, rep(0, 18 - j)))
    # isometric log-transformation of offset means
    meandat$ilrAA[, 1:17] <- ilr(offsetMean)   #
    # predicted values of the offset means     #
    fit.offset <- predict(lmAA, newdata = meandat, se.fit = TRUE)

    # calculate the estimate and half of 95% confidence interval
    estimate <- rounde(fit.offset$fit - fit.mean$fit, digits=4)
    interval <-
      rounde(
      1.96 * sqrt((
        fit.mean$df * fit.mean$se.fit ^ 2 + fit.offset$df * fit.offset$se.fit ^ 2
      ) /
        (fit.mean$df + fit.offset$df )
      ),
      digits=4)

        # predicted a value table “replaced_compo” to return
    if(j==1){
      replaced_compo <- data.frame(est = estimate, int=interval)
    } else {
```

```
    replaced_compo <- cbind(replaced_compo,
                     estimate, interval)
   }
  } # end of "for" statement

   # column names with prefix Est_ for the estimate, and Int_ for half CI
  names(replaced_compo) <- paste(rep(c("Est_", "Int_"), time=18),
                              rep(aminoacids, each=2),
                             sep="")
  return(replaced_compo)
}  # end of function "replace.compo"

# function to round digits
rounde <- function(x,digits=0) {
       expo<-10^digits
       return(ifelse(abs(x*expo) - floor(abs(x*expo)) < 0.5, sign(x*expo) *
       floor(abs(x*expo)), sign(x*expo) * (floor(abs(x*expo)) + 1))/expo)
} # end of function "rounde"

###                                            ###
### Multivariate Linear Regression Analysis    ###
###    the results are "Estimates.diff"         ###

# prepare data.frame objects
Coefficients  <- data.frame()
Estimates.diff <- data.frame()

# calculate the predicted effect of 0.1% replacement 18 times
for(i in 1:18){
  # isometric log transformation
  df_analysis %>%
    filter(flg==1) %>%                              # excluding if necessary
    acomp(aminoacids) %>%
    ilr() -> ilrAA

  # multivariate linear models
  lmAA <- df_analysis %>%
    filter(flg==1) %>%                              # excluding if necessary
    lm(
      as.numeric(glucose) ~ ilrAA + covariate1 + covariate2 + ... + covariateN ,
      data = .
      )

  # summarize coefficients of linear regression models
  Coefficients <- rbind(Coefficients,
    data.frame(
      Amino_acid = aminoacids[1],                   # variable name
      Estimate = summary(lmAA)$coefficients[2, 1],     # estimate
      Error = summary(lmAA)$coefficients[2, 2],        # standard error
      p_value = summary(lmAA)$coefficients[2, 4]       # p value
  ))
```

```
  # geometric means of amino acids
  df_analysis %>%                                        #
    filter(flg==1) %>%
    acomp(aminoacids) %>%
    apply(2, function(x) exp(mean(log(x)))) -> compMean

  # calculate replacement
   est.diff <- replace.compo(lmAA, compMean)          #

  # summarise replacement (estimate, half of 95% confidence interval)
  if(i==1){                                               # first row
    Estimates.diff <- est.diff
  } else {                                                # second and the following row
    Estimates.diff <- rbind(Estimates.diff, est.diff)
  }

  # change the order of amino acids
  aminoacids <- c(aminoacids[2:18], aminoacids[1])     # change the order of the list
  } # end of "for" statement

# the results are "Estimates.diff"
```
