# Supplementary material for: Dietary Amino Acid Composition and Glycemic Biomarkers in Japanese Adolescents
Source: Nutrients. 2024 Mar 19;16(6):882. doi: 10.3390/nu16060882 (PMC10975557; doi:10.3390/nu16060882)
Supplement: Supplementary file 1 [file nutrients-16-00882-s001.zip › Supplementary_TableS2.pdf]

Supplementary Table S2. Covariance matrix of dietary amino acids.

	ILE	LEU	LYS	MET	CYS	PHE	TYR	THR	TRP	VAL	HIS	ARG	ALA	ASP	GLU	GLY	PRO	SER
**ILE**	0.000	<0.001	0.002	0.001	0.007	0.001	0.001	0.001	0.001	<0.001	0.005	0.006	0.004	0.002	0.004	0.008	0.008	0.001
**LEU**	<0.001	0.000	0.004	0.001	0.006	0.001	0.001	0.001	0.001	<0.001	0.005	0.007	0.005	0.003	0.003	0.008	0.007	0.001
**LYS**	0.002	0.004	0.000	0.003	0.016	0.006	0.005	0.002	0.005	0.004	0.004	0.010	0.006	0.004	0.011	0.009	0.016	0.007
**MET**	0.001	0.001	0.003	0.000	0.007	0.002	0.001	0.001	0.002	0.001	0.003	0.005	0.002	0.002	0.006	0.006	0.012	0.002
**CYS**	0.007	0.006	0.016	0.007	0.000	0.003	0.005	0.007	0.004	0.006	0.011	0.004	0.006	0.006	0.005	0.007	0.014	0.003
**PHE**	0.001	0.001	0.006	0.002	0.003	0.000	<0.001	0.002	<0.001	0.001	0.006	0.005	0.004	0.002	0.002	0.007	0.007	<0.001
**TYR**	0.001	0.001	0.005	0.001	0.005	<0.001	0.000	0.001	<0.001	<0.001	0.006	0.004	0.004	0.002	0.003	0.007	0.009	0.001
**THR**	0.001	0.001	0.002	0.001	0.007	0.002	0.001	0.000	0.001	0.001	0.003	0.004	0.002	0.001	0.005	0.005	0.012	0.002
**TRP**	0.001	0.001	0.005	0.002	0.004	<0.001	<0.001	0.001	0.000	0.001	0.006	0.004	0.004	0.002	0.003	0.007	0.009	0.001
**VAL**	<0.001	<0.001	0.004	0.001	0.006	0.001	<0.001	0.001	0.001	0.000	0.006	0.006	0.005	0.002	0.003	0.008	0.007	0.001
**HIS**	0.005	0.005	0.004	0.003	0.011	0.006	0.006	0.003	0.006	0.006	0.000	0.007	0.003	0.004	0.010	0.004	0.019	0.007
**ARG**	0.006	0.007	0.010	0.005	0.004	0.005	0.004	0.004	0.004	0.006	0.007	0.000	0.002	0.002	0.010	0.002	0.022	0.005
**ALA**	0.004	0.005	0.006	0.002	0.006	0.004	0.004	0.002	0.004	0.005	0.003	0.002	0.000	0.002	0.009	0.001	0.020	0.004
**ASP**	0.002	0.003	0.004	0.002	0.006	0.002	0.002	0.001	0.002	0.002	0.004	0.002	0.002	0.000	0.007	0.004	0.016	0.002
**GLU**	0.004	0.003	0.011	0.006	0.005	0.002	0.003	0.005	0.003	0.003	0.010	0.010	0.009	0.007	0.000	0.011	0.004	0.003
**GLY**	0.008	0.008	0.009	0.006	0.007	0.007	0.007	0.005	0.007	0.008	0.004	0.002	0.001	0.004	0.011	0.000	0.025	0.007
**PRO**	0.008	0.007	0.016	0.012	0.014	0.007	0.009	0.012	0.009	0.007	0.019	0.022	0.020	0.016	0.004	0.025	0.000	0.008
**SER**	0.001	0.001	0.007	0.002	0.003	<0.001	0.001	0.002	0.001	0.001	0.007	0.005	0.004	0.002	0.003	0.007	0.008	0.000

ILE, Isoleucine; LEU, Leucine; LYS, Lysine; MET, Methionine; CYS, Cysteine; PHE, Phenylalanine; TYR, Tyrosine; THR, Threonine; TRP, Tryptophan; VAL, Valine; HIS, Histidine; ARG, Arginine; ALA, Alanine; ASP, Aspartic acid; GLU, Glutamic acid; GLY, Glycine; PRO, Proline; SER, Serine. The smaller the value is, the more a pare is correlated.
