# Supplementary material for: Dietary Amino Acid Composition and Glycemic Biomarkers in Japanese Adolescents
Source: Nutrients. 2024 Mar 19;16(6):882. doi: 10.3390/nu16060882 (PMC10975557; doi:10.3390/nu16060882)
Supplement: Supplementary file 1 [file nutrients-16-00882-s001.zip › Supplementary_TableS3.pdf]

Supplementary Table S3. Simple regression analysis of nutrients for glucose, and log-transformed insulin.

	Glucose, mg/dl			Log(insulin, μU/ml)		
	**Estimate**	**SE**	**p**	**Estimate**	**SE**	**p**
**Energy, kcal**	6.61×10^{-4}	2.50×10^{-4}	0.008	-3.54×10^{-5}	2.05×10^{-5}	0.084
**Protein, %E**	-0.173	0.069	0.012	-3.58×10^{-3}	5.65×10^{-3}	0.527
**TDF, g**	-0.373	0.110	<0.001	0.0142	0.0090	0.116
**SFA, %E**	0.073	0.065	0.257	-8.05×10^{-3}	5.27×10^{-3}	0.127
**Glycemic load, %**	0.013	0.007	0.066	-1.69×10^{-4}	5.79×10^{-4}	0.771

SE, standard error; TDF, total dietary fiber; SFA, saturated fatty acids. Insulin was natural log transformed.
