# Supplementary material for: Dietary Amino Acid Composition and Glycemic Biomarkers in Japanese Adolescents
Source: Nutrients. 2024 Mar 19;16(6):882. doi: 10.3390/nu16060882 (PMC10975557; doi:10.3390/nu16060882)
Supplement: Supplementary file 1 [file nutrients-16-00882-s001.zip › Supplementary_TableS4.pdf]

Supplementary Table S3. Multivariate linear regression analysis for amino acid group composition.

	Geometric Mean, %	Covariance matrix				Glucose, mg/dl		log(Insulin, μU/ml)		log(HOMA-IR)		log(HOMA-β, %)	
						β (SE)	p	β (SE)	p	β (SE)	p	β (SE)	p
**BCAAs**	18.18	0	<0.001	0.001	0.001	-15.03 (13.95)	0.282	-0.58 (1.06)	0.585	-0.77 (1.12)	0.495	0.24 (1.01)	0.81
**AAAs**	9.54	<0.001	0	0.001	0	9.63 (11.12)	0.387	0.96 (0.84)	0.257	1.08 (0.89)	0.226	0.37 (0.81)	0.651
**SAAs**	4.01	0.001	0.001	0	0.001	6.35 (10.43)	0.543	-0.13 (0.79)	0.867	-0.07 (0.84)	0.929	-0.25 (0.76)	0.739
**Other AAs**	68.25	0.001	<0.001	0.001	0	-0.95 (11.5)	0.934	-0.25 (0.87)	0.778	-0.24 (0.92)	0.793	-0.36 (0.83)	0.669

Insulin, homeostatic model assessment for insulin resistance (HOMA-IR) and for beta cell function (HOMA-β) were natural log transformed. ILR; isometric log-transformed; β, coefficient; SE, standard deviation; BCAAs, blanched-chain amino acids such as isoleucine, leucine, and valine; AAAs: aromatic amino acids such as phenylalanine, tyrosine, and tryptophan; SAAs: sulfur amino acids such as methionine, and cysteine; other AAs such as lysine, threonine, histidine, arginine, alanine, aspartic acid, glutamic acid, glycine, pro-line, and serine. Regression coefficients are for the first term relative to the other terms in ILR-compositions. Multivariate regression models were adjusted for age, sex, z-score of body mass index, physical activity, sleep duration, screen time, single parents, passive smoking, siblings, energy, protein, and total dietary fiber.
